# Sanatoria and Their Critics

**Published:** 1920-01-24

**Authors:** 


### SANATORIA AND THEIR CRITICS.

In the very white paper and very clear type of Hansard we notice that a noble lord asked Dr. Addison how the death-rate for consumption had been going. The Minister of Health replied that in 1910 it was 1.015; in 1913, 1.004; in 1918—civilians only—1.343. The reply contains a fact well known to most intelligent persons, which is that the hardships of war quickly increased disease. However, the layman who wants to criticise will point to all the money spent since the Insurance Act came in in 1913, and ask if the result can be considered satisfactory; and, if not, in what particulars improvement can be made. The easiest point of attack is the sanatorium, and we notice indications in the daily Press that it has been selected again. Most of the people who write letters to newspapers take extreme views, and, as the modern popular journalist mirrors his audience whenever he can, so as to please them and make them docile, where he wants them docile, it is not surprising to meet with the attitude that the sanatorium is, as John Davidson said of another and older institution, "a lifeboat warped and sunk." Now, quite dispassionately, we say that it cannot be that. If it were, it would not have lived and grown for twenty years. Most remedies, particularly remedies for consumption, are carried out of sight and mind by the stream of time. But the sanatorium, though abused, steadfastly remains; and the reason it remains is that it *is* a lifeboat after all—not a quick lifeboat by any means, making very few trips; in fact, a terribly slow one; not a secure one, for it tumbles out most of its passengers. But, with all deductions, it is the only lifeboat, the only chance of safety. Those who criticise it hardest would be very likely to hurry for a seat in it were they pronounced consumptive, just as the anti-vaccinators in the classical epidemic went and got vaccinated of their own accord. Some of its lack of efficiency can be remedied. Here is one point, in a letter from a solicitor to a daily paper, in which he says that 90 per cent. of his fellow-patients broke the rules. Unchecked, the consumptive at a public sanatorium always does, just as he always grumbles at the food. He wants a good medical superintendent over him, to supervise him and establish a tone of public opinion that makes for righteousness. But good doctors are chary of taking charge of public sanatoriums unless forced to by lack of health, because the position is robbed of all means of securing respect and authority. For the last two or three years consumptive soldiers have been defying matrons left in charge of the sanatoriums, breaking out, getting drunk, breaking out to play football even, and then getting discharged to hang round War Pensions Committees. There is no use in cloaking these things, and responsible laymen should know where the fault lies.

Again, another great and inherent fault of the sanatorium *régime* is that it takes so long to effect an arrest that will stand that the required length of stay is impossible owing to the numbers to be treated. The residential colony for consumptives is an answer to that objection, but the colony needs supplementing for the same reason, for the number of patients is now vast. Therefore every public sanatorium should have a thriving outdoor department, where its superintendent can employ picked ex-patients. Every healthy post about a sanatorium, including those of the nurses and matron, should be filled by an ex-patient. Were such reforms as these, which do not call for subscription-lists and impossible demands for building, put in hand, we might hear less from our lay friends in derogation of British sanatoriums.

